# Supplementary material for: The sex-specific factor SOA controls dosage compensation in Anopheles mosquitoes
Source: Nature. 2023 Sep 28;623(7985):175–82. doi: 10.1038/s41586-023-06641-0 (PMC10620080; doi:10.1038/s41586-023-06641-0)
Supplement: Supplementary file 1 — Supplementary Fig. 1, Supplementary Note 1, Supplementary Tables 4 and 5 and legends for Supplementary Tables 1–3. [file 41586_2023_6641_MOESM1_ESM.pdf]

nature portfolio

https://doi.org/10.1038/s41586-023-06641-0

# Supplementary information

# The sex-specific factor SOA controls dosage compensation in *Anopheles* mosquitoes

In the format provided by the authors and unedited

# The sex-specific factor SOA controls dosage compensation in *Anopheles* mosquitos

Agata Izabela Kalita[1], Eric Marois[2,6], Magdalena Kozielska[3], Franz J. Weissing[3], Etienne Jaouen[2], Martin M. Möckel[1], Frank Rühle[1], Falk Butter[1,4], M. Felicia Basilicata[1,5,6], Claudia Isabelle Keller Valsecchi[1,6]*

**Affiliations**

[1]Institute of Molecular Biology (IMB); Mainz, Germany.

[2]Inserm U1257, CNRS UPR9022, Université de Strasbourg; Strasbourg, France.

[3]Groningen Institute for Evolutionary Life Sciences, University of Groningen; Groningen, Netherlands.

[4]Institute of Molecular Virology and Cell Biology, Friedrich Loeffler Institute, 17493 Greifswald, Germany.

[5]Institute of Human Genetics, University Medical Center of the Johannes Gutenberg University Mainz, Mainz, Germany.

[6]equal contribution

*Corresponding author. Email: c.keller@imb-mainz.de

Supplementary Figure 1

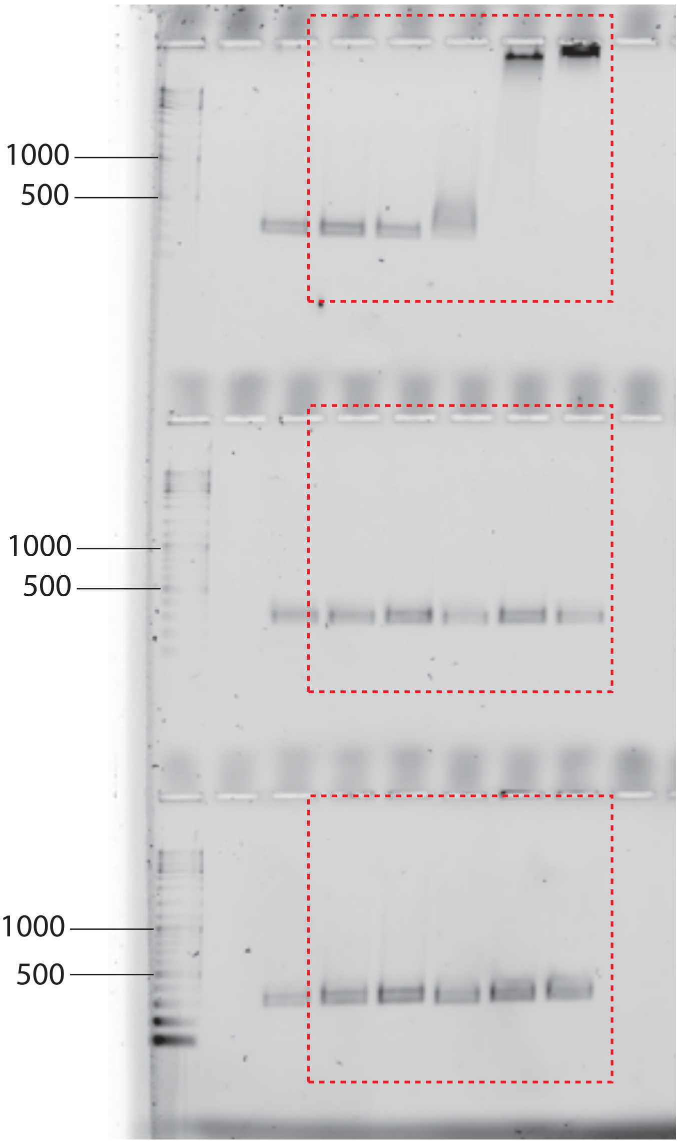


2023-05-12 emsa 300 bp -[SYBR Safe].tif

Extended Data Fig. 8j

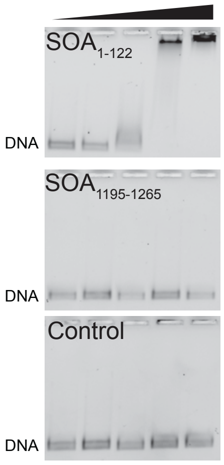

Extended Data Fig. 8k

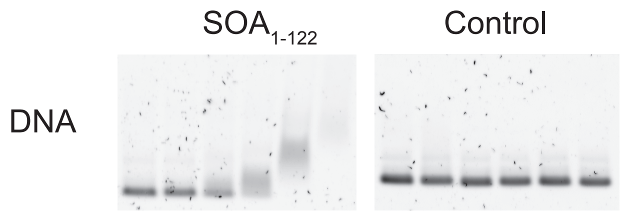


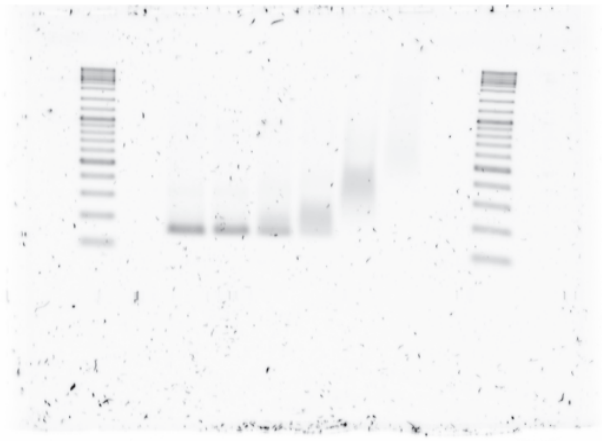

20210430_AF647K-SOA-dna-sybr-600-[SYBR Safe].tif

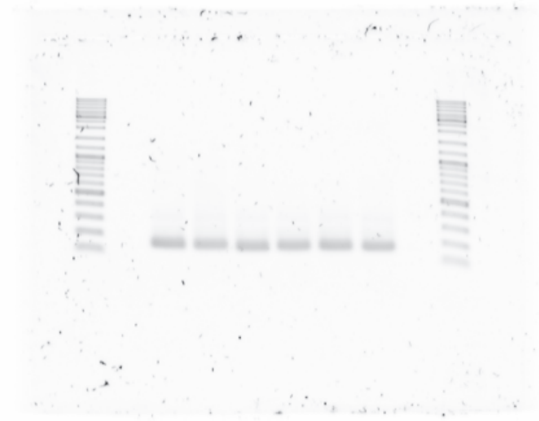

20210501_GST-ctrl-DNA-sybr-[SYBR Safe].tif

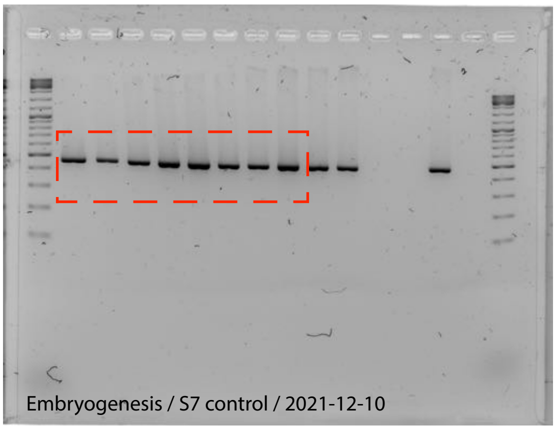


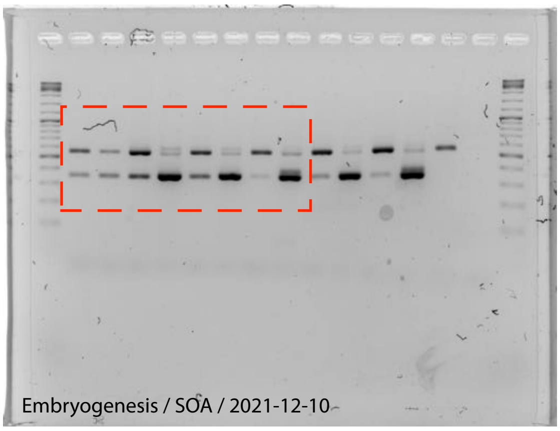


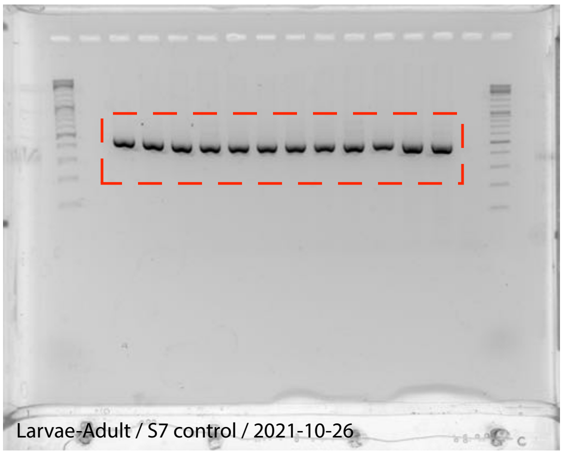


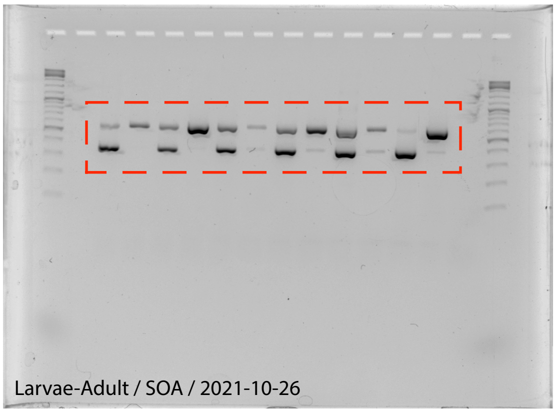


## Extended Data Fig. 4b

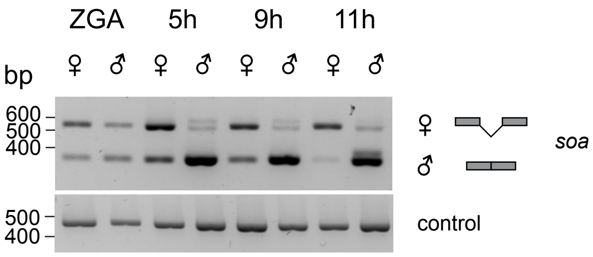


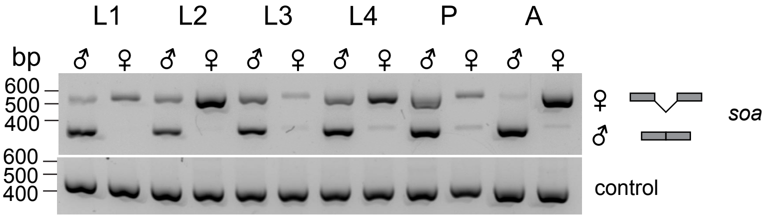

G20 Lab 2023-05-11 13h09m36s(Chemiluminescence).jpg

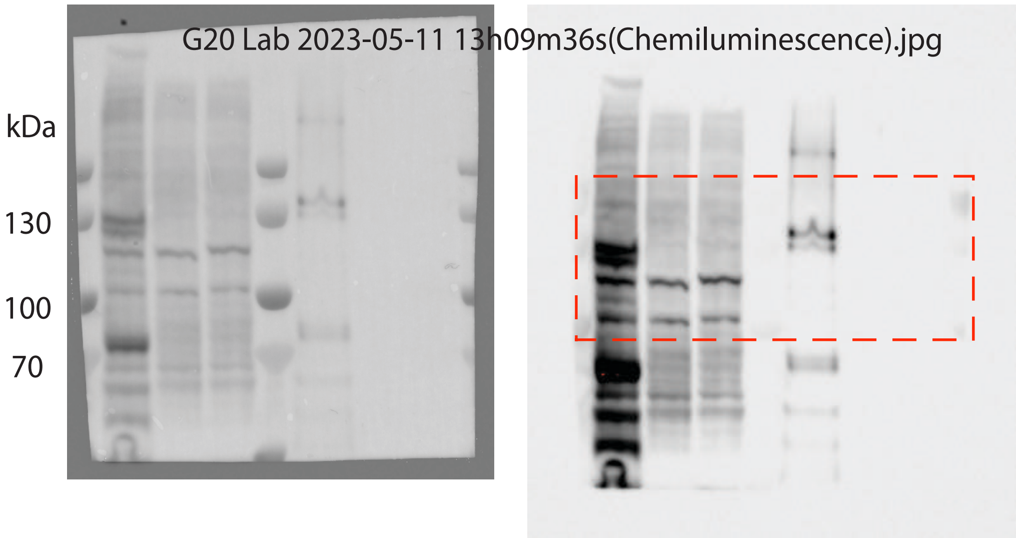


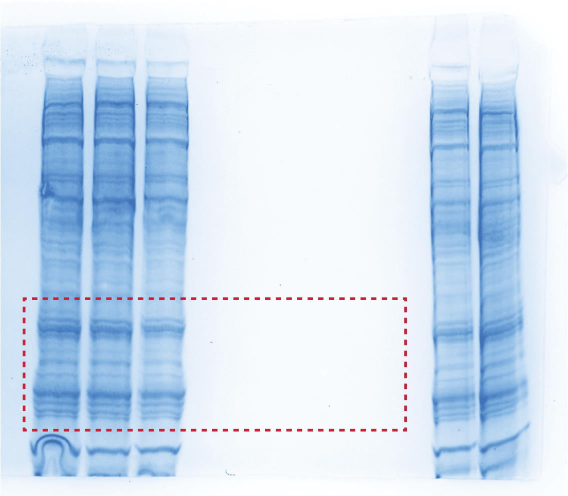

G20 Lab 2023-05-11 14h11m25s.tif

## Extended Data Fig. 5b

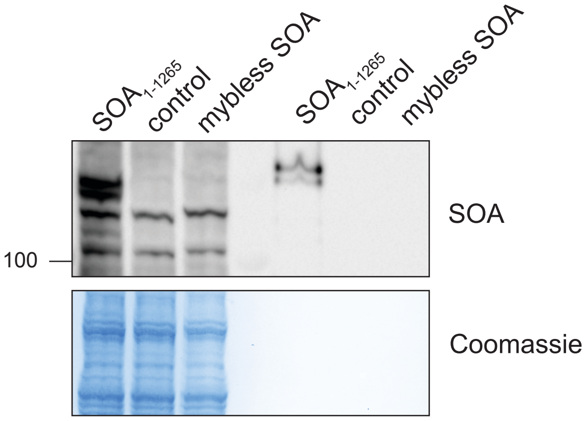


SOA + ladder merged

SOA

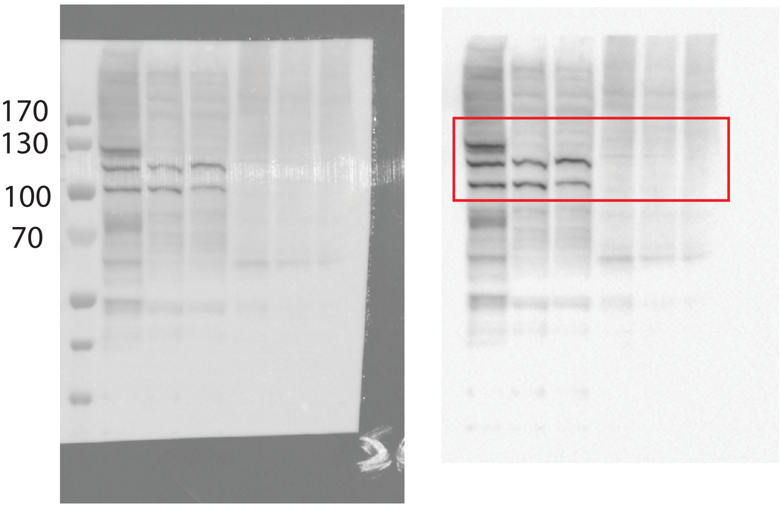


G20 Lab 2023-05-05 13h12m29s(Chemiluminescence).tif

Pol2 (ladder merged)

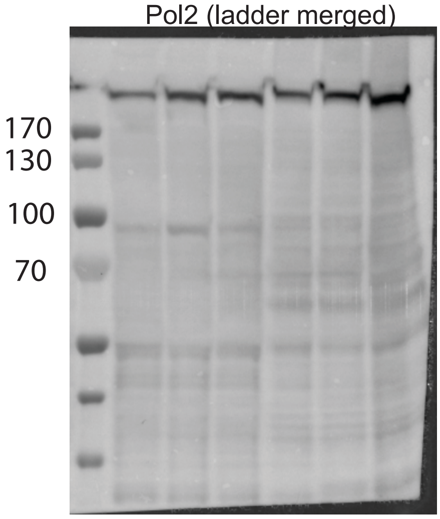


G20 Lab 2023-05-09 17h29m41s+G20 Lab 2023-05-09 17h13m44s.jpg

## Extended Data Fig. 5a

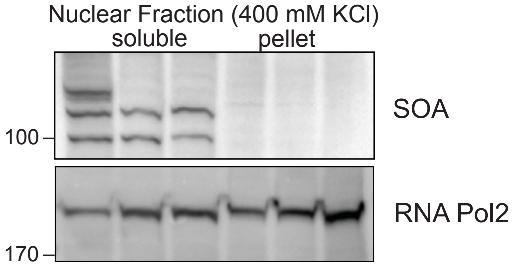

## Extended Data Fig. 8i

G20 Lab 2021-06-15 01h36m11s.tif

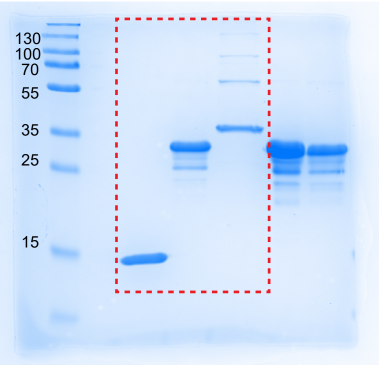


## Extended Data Fig. 8h

G20 Lab 2023-05-12 20h10m12s.tif

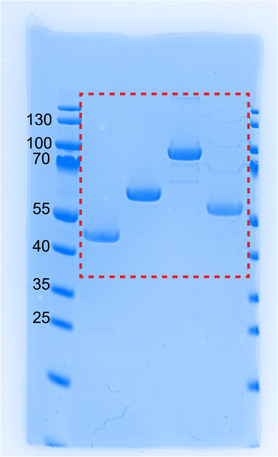


## Extended Data Fig. 9a

G20 Lab 2022-05-23 10h34m58s(SYBR Safe).jpg

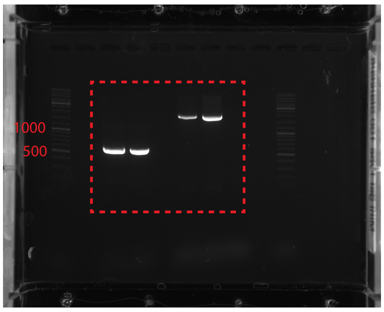


G20 Lab 2022-03-10 02h29m46s(Chemiluminescence).tif

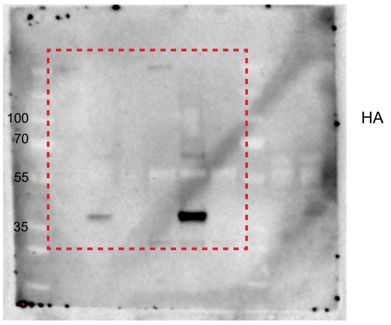


H3 G20 Lab 2022-03-11 06h49m42s+G20 Lab 2022-03-11 06h46m01s

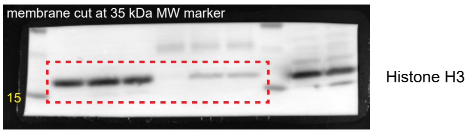


## Extended Data Fig. 5c

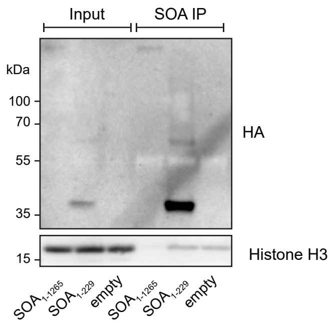

# Supplementary Note

## Evolution of the *SOA* gene

*SOA* evolved by a gene duplication event from its paralogue *AGAP005747* (Extended Data Fig. 1b-e), which is located directly next to *SOA* (Extended Data Fig. 1e). The region encoding *SOA* is syntenic among Culicidae and, except for *SOA*, maintained between the Anophelinae and Culicinae subfamilies (Extended Data Fig. 1e). *AGAP005747* mRNA is maternally deposited and its expression is not sex-biased (Extended Data Fig. 1f). *SOA* instead is not maternally provided, but strongly induced after ZGA (Fig. 1b) and maintains male-biased expression throughout all subsequent developmental stages (Extended Data Fig. 1g), indicating that after the duplication, SOA diverged from the function of its paralogue.

The *SOA* paralogue (*AGAP005747*) differs from SOA in lacking the N-terminal myb-DNA binding domain (Extended Data Fig. 3a-b). The intronic stop codon and exon-intron junctions are fully conserved among *A. gambiae, A. arabiensis, A. minimus,* and *A. albimanus* (Extended Data Fig. 4c) indicating a strong selection to maintain full-length SOA expression only in males, but not females

## Supplementary discussion

In this study, we have identified and characterized the gene *SOA*, which encodes the master regulator of *Anopheles gambiae* DC. *SOA* evolved in *Anopheles* and displays conserved, sex-specific alternative splicing. It is a DNA binding protein, binds to X-linked gene promoters and is sufficient to induce X-chromosome upregulation upon expression in female cells and mosquitos. Its absence *in vivo* leads to a male-specific developmental delay linked to the dysregulation of the X chromosome. Thus, SOA is the first master regulatory factor of a chromosome-wide DC mechanism described in a non-model organism.

*Sex determination cascades and alternative splicing of SOA.*

The fact that full-length SOA expression in females is prevented by alternative splicing is conceptually similar to the regulation of *Drosophila msl-2* [1]. The female sex determination factor SXL binds to an alternatively spliced intron preventing *msl-2* RNA export and translation. Thereby, MSL2 protein is only present in *Drosophila* males. In contrast to MSL2, peptides of the truncated *Anopheles* female SOA protein are detectable in mass spectrometry. However, female $SOA_{1\text{-}229}$ does not associate with the X chromosome and is not functional for DC.

At ZGA, *SOA* isoforms are identical between sexes. Shortly thereafter, *SOA* sex-specific splicing is progressively established. We therefore hypothesize that a female factor already present in the egg prevents intron 2 excision. The sex determination pathway factor Femaleless (Fle) contains several RNA-binding domains and its knock-

down is associated with misregulation of X-linked transcripts in females [2]. Therefore, Fle could be preventing *SOA* splicing in females akin to SXL targeting *msl-2*.

*SOA* evolved by a tandem gene duplication event from a paralogue, which is not sex-specifically spliced. This raises the question of where SOA's intron comes from. *SOA* may have hijacked intron sequences from conserved genes with sex-specific alternative splicing. This would make the evolution of the splicing mechanism more rapid, as the sequence could take advantage of pre-existing splicing factors. Indeed, FLE controls the sex-specific splicing of several transcripts (e.g. *fruitless* or *doublesex* [2]), which are well conserved among insects [3]. If gene duplication precedes the evolution of alternative splicing, a newly arisen DC factor such as SOA would be expected to be beneficial in only one sex, but detrimental in the other one, since it will lead to the overexpression of X-linked genes. Under these conditions, alternative splicing is strongly selected, as it may alleviate or even resolve the conflict, whereupon DC can spread to fixation.

The phenotype of *SOA-KI* mosquitos is different from mutants in the sex determination pathway, which show sex reversal, sterility or lethality of variable penetrance [2,4,5]. *Yob* knock-down causes a skewed sex ratio, but its impact on developmental timing and X chromosome expression in males has not been assessed [4]. Conversely, ectopic expression of *Yob* in females leads to different phenotypes including developmental delay, intersex phenotypes and low penetrance lethality [4,6]. The expression of *Guy1*, the Y-linked maleness gene in *Anopheles stephensi*, confers complete female-specific lethality at embryonic stages, which is accompanied by an upregulation of X-linked genes [7]. The molecular functions of Guy1/Yob are not known yet, but our data shows that SOA directly binds to the X chromosome, while interfering with its function is not lethal. We favor a model where *Guy1/Yob* induce *SOA*, but also other yet to be identified factors, the latter of which or their combination with X-misregulation is causal to sex-specific lethality. It will be interesting to assess *Guy1*-mediated lethality in *A. stephensi* under conditions where SOA is not functional.

*Specificity and pattern of X chromosome binding.*

By which molecular mechanism can SOA identify the X chromosome? SOA's property of targeting promoters of active genes is different from e.g. the *Drosophila* MSL complex, which initially binds at high affinity sites and then spreads to X-linked genes [8]. The SOA-bound promoters are enriched in CA-dinucleotide repeat sequences, which became specifically expanded in the X chromosome of *Anopheles*, but not in the related *Aedes* mosquitos that lack sex chromosomes. The CA-expansion in *Anopheles* may have occurred in a similar fashion as for the *Drosophila miranda* X chromosome, where the domestication of a mutant helitron transposable element has contributed to expansion of GA repeats for MSL binding [9]. Several features of the X-linked CA motifs (higher frequency, increased length and motif clustering) may provide cooperativity and thereby be relevant to provide stable chromatin association of SOA. For *Drosophila* GAGA factor (as SOA, a DNA-binding - BTB domain containing protein), cooperative binding provides recognition of the proper target sites, despite

the relatively high abundance of individual GAGA-motifs across the genome [10]. Our data shows that the BTB-domain boosts SOA's ability to bind DNA. However, the isolated myb - BTB fragment is not sufficient to distinguish CA- from non-CA sequences *in vitro*. Because the myb-domain is necessary for X chromosome binding *in vivo* we propose that allosteric regulation or co-factor recruitment provided by the C-terminal part help SOA to find its proper target sites.

By directly associating with the X chromosome SOA joins a very small list of master regulators that are sufficient to induce chromosome-wide expression alterations (*D. melanogaster* MSL2 [11], *C. elegans* SDC-2 [12], Mammalian *Xist* [13]). What happens next when SOA is recruited to the X? Since SOA does not have an enzymatic domain that could directly influence the chromatin state by e.g. catalyzing histone modifications, it is likely that other factors interact with SOA. Akin to *Drosophila* MOF, which is expressed in both sexes, these SOA co-factors do not necessarily need to be sex-specific, as SOA expression in females triggers upregulation of X-linked genes without *Yob*. After SOA recruitment to X-linked promoters, transcription itself (e.g. pause release or elongation [14]) or co-transcriptional RNA processing events [15] may be altered. The associated mechanisms that superimpose on gene regulation *per se* remain to be identified.

*Role of SOA in DC and physiological consequences of its loss.*

In contrast to the lethal phenotypes in model organisms, the loss of DC in males or its ectopic induction in females is associated with a developmental delay in *Anopheles*. This is interesting for several reasons. While the molecular activities of DC complexes in model organisms have been studied in great detail, the physiological consequences of their absence and especially the reasons for lethality still remain unclear. Hypotheses range from misregulation of very few, putative haplo-lethal genes encoded on the X, to a global gene-dosage imbalance causing perturbation of gene regulatory networks, overload of cellular machineries such as the ribosome, chaperones and proteotoxicity [16]. In this dosage-imbalance model, the nature of the X-linked genes is not the primary determinant of lethality. Instead, lethality is caused by the extent of the imbalance and related to the number of X-linked genes and their interaction partners on autosomes [17]. Despite having comparable overall gene numbers, *Drosophila* possesses approximately 2500 X-linked protein-coding genes, while *Anopheles* has only 1063. It is also noteworthy that a lack of DC in *Drosophila* is not fully incompatible with development: expression imbalance in *msl*-mutants manifests as early as a few hours of embryogenesis [18]. However, lethality only occurs at the larval/early pupal stage around 6 days later [19] and notably, rare escapers reaching the adult stage can be observed in *roX1/2* mutants [20]. Another factor possibly responsible for the weaker phenotypic consequences in *Anopheles* is the generation of autosomal retrocopies of X-linked genes [21]. Thereby, dosage-sensitive genes can "escape" the X chromosomal imbalance and there is no need for DC anymore. Nonetheless, the developmental delay, as shown by computational modeling and supported by experimental observations in our laboratory populations, is a strong

enough phenotype to provide sufficient evolutionary pressure for DC to evolve. The fitness defect may be even more relevant in a natural environment, where female *Anopheles* mate only once with a chosen "significant other" in swarms of up to a few hundred males. Given the strongly skewed sex ratio in these mating swarms, a developmental delay of several hours may become very relevant to the reproductive success of a given male [22].

It has remained a conundrum why many species with heteromorphic sex chromosomes (e.g. birds) do not exhibit chromosome-wide DC [17,23]. Our data shows that non-essentiality may permit the evolution of a DC master regulator despite being beneficial for one sex, but reducing the fitness of the other one. In this scenario of sexual conflict, our model predicts that a gene such as *SOA* can be present as a polymorphism, where only some individuals in the population exhibit DC. This underscores the importance of studying this mechanism with sufficient sampling rate, as DC alleles could be rare among populations. It will be crucial to perform future studies in natural contexts and in species with different sex determination systems, extent of sexual dimorphism and reproductive strategies. Finally, we note that exploiting X chromosome misregulation has been proposed to artificially generate single-sex populations or sex ratio distortion gene drives for vector control programs [6,7]. Our discovery that induction of the SOA-DC pathway - at least under the conditions studied by us - is not strongly detrimental for females, warrants further studies to uncover factors and mechanisms underlying sex-specific lethality to eventually harness them in malaria vector control programs.

## References


1. Beckmann, K., Grskovic, M., Gebauer, F. & Hentze, M. W. A dual inhibitory mechanism restricts msl-2 mRNA translation for dosage compensation in Drosophila. *Cell* **122**, 529–540 (2005).
2. Krzywinska, E. *et al.* femaleless Controls Sex Determination and Dosage Compensation Pathways in Females of Anopheles Mosquitoes. *Curr. Biol.* **31**, 1084–1091.e4 (2021).
3. Price, D. C., Egizi, A. & Fonseca, D. M. The ubiquity and ancestry of insect doublesex. *Sci. Rep.* **5**, 13068 (2015).
4. Krzywinska, E., Dennison, N. J., Lycett, G. J. & Krzywinski, J. A maleness gene in the malaria mosquito Anopheles gambiae. *Science* **353**, 67–69 (2016).
5. Kyrou, K. *et al.* A CRISPR–Cas9 gene drive targeting doublesex causes complete

population suppression in caged Anopheles gambiae mosquitoes. *Nat. Biotechnol.* **36**, 1062 (2018).

6. Krzywinska, E. & Krzywinski, J. Effects of stable ectopic expression of the primary sex determination gene Yob in the mosquito Anopheles gambiae. *Parasit. Vectors* **11**, 648 (2018).
7. Qi, Y. *et al.* Guy1, a Y-linked embryonic signal, regulates dosage compensation in Anopheles stephensi by increasing X gene expression. *Elife* **8**, (2019).
8. Straub, T., Zabel, A., Gilfillan, G. D., Feller, C. & Becker, P. B. Different chromatin interfaces of the Drosophila dosage compensation complex revealed by high-shear ChIP-seq. *Genome Res.* **23**, 473–485 (2013).
9. Ellison, C. E. & Bachtrog, D. Dosage compensation via transposable element mediated rewiring of a regulatory network. *Science* **342**, 846–850 (2013).
10. Tang, X. *et al.* Kinetic principles underlying pioneer function of GAGA transcription factor in live cells. *Nat. Struct. Mol. Biol.* **29**, 665–676 (2022).
11. Kelley, R. L. *et al.* Expression of msl-2 causes assembly of dosage compensation regulators on the X chromosomes and female lethality in Drosophila. *Cell* **81**, 867–877 (1995).
12. Dawes, H. E. *et al.* Dosage compensation proteins targeted to X chromosomes by a determinant of hermaphrodite fate. *Science* vol. 284 1800–1804 (1999).
13. Brockdorff, N. *et al.* The product of the mouse Xist gene is a 15 kb inactive X-specific transcript containing no conserved ORF and located in the nucleus. *Cell* **71**, 515–526 (1992).
14. Ferrari, F. *et al.* 'Jump start and gain' model for dosage compensation in Drosophila based on direct sequencing of nascent transcripts. *Cell Rep.* **5**, 629–636 (2013).
15. Rücklé, C. *et al.* RNA stability controlled by m6A methylation contributes to X-to-autosome dosage compensation in mammals. *Nat. Struct. Mol. Biol.* (2023) doi:10.1038/s41594-023-00997-7.
16. Lee, H. *et al.* Effects of Gene Dose, Chromatin, and Network Topology on Expression in

Drosophila melanogaster. *PLoS Genet.* **12**, e1006295 (2016).

17. Basilicata, M. F. & Keller Valsecchi, C. I. The good, the bad, and the ugly: Evolutionary and pathological aspects of gene dosage alterations. *PLoS Genet.* **17**, e1009906 (2021).
18. Samata, M. *et al.* Intergenerationally Maintained Histone H4 Lysine 16 Acetylation Is Instructive for Future Gene Activation. *Cell* (2020) doi:10.1016/j.cell.2020.05.026.
19. Belote, J. M. & Lucchesi, J. C. Male-specific lethal mutations of Drosophila melanogaster. *Genetics* **96**, 165–186 (1980).
20. Kim, M., Faucillion, M.-L. & Larsson, J. RNA-on-X 1 and 2 in Drosophila melanogaster fulfill separate functions in dosage compensation. *PLoS Genet.* **14**, e1007842 (2018).
21. Miller, D. *et al.* Retrogene Duplication and Expression Patterns Shaped by the Evolution of Sex Chromosomes in Malaria Mosquitoes. *Genes* **13**, (2022).
22. Smidler, A. L., Scott, S. N., Mameli, E., Shaw, W. R. & Catteruccia, F. A transgenic tool to assess Anopheles mating competitiveness in the field. *Parasit. Vectors* **11**, 651 (2018).
23. Furman, B. L. S. *et al.* Sex Chromosome Evolution: So Many Exceptions to the Rules. *Genome Biol. Evol.* **12**, 750–763 (2020).

# Supplementary Table 4. Antibodies

| Antibody target | Species | Source | Cat. No. | Application | Dilution |
|---|---|---|---|---|---|
| SOA | Rabbit | Custom (Eurogentec), epitope-purified by the IMB PPCF<br>Epitope: SOA amino acids 1-122 (recombinant, purified in E. *coli*) | N.A. (Rabbit 87), #540887-220 62021 | IF | 1:300 |
| | | | | Western | 1:1000 |
| | | | | CUT&Tag | 1:50 |
| | | | | IP | 3-4 µL per IP |
| HA.11 | Mouse | Biolegend | BLD-901502 | CUT&Tag | 1:50 |
| | | | | Western | 1:2000 |
| Histone H3 | Rabbit | Cell Signalling | 9715S | Western | 1:4000 |
| Histone H3 (mAb) | Mouse | Active Motif | 39763 | IF | 1:400 |
| RNA pol II antibody (mAb) | Mouse | Active Motif | 39097 | Western | 1:5000 |
| | | | | IF | 1:400 |
| RNA pol II CTD phospho Ser2 antibody (mAb) | Rat | Active Motif | 61984 | IF | 1:300 |
| phospho H3 (S10) | Mouse IgG2b, κ | Biolegend | 650801 | IF | 1:400 |
| IgG control | Rabbit | Abcam | ab37415 | CUT&Tag | 1:50 |
| αMs IgG | Rabbit | Abcam | ab6709 | CUT&Tag | 1:100 |
| αRb IgG | Guinea pig | Sigma-Aldrich | SAB3700890 | CUT&Tag | 1:100 |
| αRb IgG coupled to AF555 | Goat | ThermoFisher | A21430 | IF | 1:400 |
| α-Mouse IgG (H+L) | Goat (HRP conjugate) | Jackson ImmunoResearch | JIM-715-035-150 | Western | 1:5000 |
| α-Rabbit IgG (H+L) | Donkey (HRP conjugate) | Jackson ImmunoResearch | JIM-711-035-152 | Western | 1:5000 |

# Supplementary Table 5. Primers

| Application | Name | Sequence | Target | Ensembl-ID | Amplicon size |
|---|---|---|---|---|---|
| qPCR | q005 | GCTATGATAAACTCGCTCCCAA | *Rp49* | *AGAP002122* | 189 bp |
| | q006 | TCATCAGCACCTCCAGCTC | | | |
| | q185 | GGAGGCGAATTTCGAACGATG | *SOA* | *AGAP005748* | 74 bp |
| | q186 | GGCCGAGATGAAGTAGGACG | | | |
| | q412 | TCTCACTATTTCCCAGAAACGA | SOA mRNA with retained intron 2 | *AGAP005748* | 121 bp |
| | q413 | GCGAAGTACGGGCTAAACGT | | | |
| | q417 | GCATACCGATCGTTTTTGCAC | SOA mRNA with excised intron 2 | *AGAP005748* | 70 bp |
| | rt013 | TGAAGCAGAGCGCGTATCAG | | | |
| | q447 | CGCACGTGGCAAAGCATAA | SOA-R transgene | *AGAP005748* | 115 bp |
| | q448 | ATGTTTCAGGTTCAGGGGGAG | | | |
| RT-PCR | rt001 | GGCGATCATCATCTACGTGC | *S7* | *AGAP010592* | 460 bp |
| | rt002 | GTAGCTGCTGCAAACTTCGG | | | |
| | rt015 | ACAGGAGATGGTGGTTCCGT | *SOA* | *AGAP005748* | canonical: 356 bp, female intron retention: 556 bp |
| | rt016 | CATCGTCATTGCAAACCAGCA | | | |
| Genotyping PCR | p102 | GACAGAAACCTTAGCAACG | *SOA-KI* | *AGAP005748* | 2026 bp with a knock-in/ 623 bp WT |
| | p103 | TCCTCGGTGCGAAAGTAGC | | | |
| Plasmid generation | s047 | TCAACTAATTTTAACCGCCTTTCGGAACATCA | *EF1α* promoter | *AGAP007406* | 975 bp (underlined: FseI restriction site) |
| | s048 | ATATAGGGCCGGCCACGAACAAAAGAAGGAAGAAAATGGCTGG | | | |
| CUT&See | Tn5MErev | [phos]CTGTCTCTTATACACATCT | - | - | - |
| | Tn5ME-A-ATTO488 | 5’ATTO$^{488}$-TCGTCGGCAGCGTCAGATGTGTATAAGAGACAG | - | - | - |
| | Tn5ME-B-ATTO488 | 5’ATTO$^{488}$-GTCTCGTGGGCTCGGAGATGTGTATAAGAGACAG | - | - | - |

## Legends of Supplementary Tables 1-3

**Supplementary Table 1 (Excel File).**

1. Pairwise Patristic Distances Between SOA and SOA paralogue sequences
2. SOA isoform read counts in embryonic stages from mRNA seq
3. SOA mRNA isoform quantification by qPCR in postembryonic stages
4. Mass Spectrometry Results of SOA Immunoprecipitation at 600 mM NaCl (SOA IP vs. IgG control IP in male extracts)
5. Mass Spectrometry Results of SOA Immunoprecipitation at 250 mM NaCl (SOA IP in males versus female extracts)
6. Fluorescence Polarization Data for recombinant SOA fragments
7. SOA-KI allele frequency in mixed population
8. Probe Sequences used in EMSA & RNA FISH
9. Sequence of the pDSAR-SOAki plasmid used to generate the SOA-KI mutant line
10. Sequence of the SOA-KI insertion in its genomic context
11. Sequence of the SOA-R locus (integration of rescue plasmid)

**Supplementary Table 2 (Excel File).** Lists of DESeq2 RNA-seq results and DiffBind CUT&Tag results.

1. List of significant peaks (FDR <0.05) of SOA in wild-type male vs wild-type female CUT&Tag
2. List of significant peaks (FDR <0.05) of SOA in *SoaKI* male vs wild-type male CUT&Tag
3. List of significant peaks (FDR <0.05) of SOA in *SOA-R* female vs wild-type female CUT&Tag
4. List of significant peaks (FDR <0.05) of SOA in *SoaKI* female vs wild-type female CUT&Tag
5. List of significant peaks (FDR <0.05) of SOA in long SOA vs empty Ag55 CUT&Tag
6. DESeq2 results table for male *SOA-KI* pupae versus wild-type male pupae (RNA-Seq)
7. DESeq2 results table for female *SOA-R* pupae versus wild-type female pupae (RNA-Seq)
8. DESeq2 results table for long vs short isoform of SOA ectopically expressed in Ag55 cells (RNA-Seq)
9. DESeq2 results table for short isoform of SOA ectopically expressed in Ag55 cells vs cells infected with an empty baculovirus (RNA-Seq)

**Supplementary Table 3 (Excel File).** Details on statistics, individual data points and median log2FC underlying figures.

1. Median log2FC values, replicate numbers, and other statistical values for embryogenesis RNA-seq
2. Median values and calculated fold changes underlying all violin plots.
