# Supplementary material for: The sex-specific factor SOA controls dosage compensation in Anopheles mosquitoes
Source: Nature. 2023 Sep 28;623(7985):175–82. doi: 10.1038/s41586-023-06641-0 (PMC10620080; doi:10.1038/s41586-023-06641-0)
Supplement: Supplementary file 2 — Reporting Summary [file 41586_2023_6641_MOESM2_ESM.pdf]

Corresponding author(s): Keller Valsecchi, Claudia Isabelle

Last updated by author(s): Aug 14, 2023

Reporting Summary

Nature Portfolio wishes to improve the reproducibility of the work that we publish. This form provides structure for consistency and transparency in reporting. For further information on Nature Portfolio policies, see our Editorial Policies and the Editorial Policy Checklist.

Statistics

For all statistical analyses, confirm that the following items are present in the figure legend, table legend, main text, or Methods section.

n/a	Confirmed	
☐	☒	The exact sample size (*n*) for each experimental group/condition, given as a discrete number and unit of measurement
☐	☒	A statement on whether measurements were taken from distinct samples or whether the same sample was measured repeatedly
☐	☒	The statistical test(s) used AND whether they are one- or two-sided<br>*Only common tests should be described solely by name; describe more complex techniques in the Methods section.*
☒	☐	A description of all covariates tested
☐	☒	A description of any assumptions or corrections, such as tests of normality and adjustment for multiple comparisons
☐	☒	A full description of the statistical parameters including central tendency (e.g. means) or other basic estimates (e.g. regression coefficient) AND variation (e.g. standard deviation) or associated estimates of uncertainty (e.g. confidence intervals)
☐	☒	For null hypothesis testing, the test statistic (e.g. *F*, *t*, *r*) with confidence intervals, effect sizes, degrees of freedom and *P* value noted<br>*Give P values as exact values whenever suitable.*
☒	☐	For Bayesian analysis, information on the choice of priors and Markov chain Monte Carlo settings
☒	☐	For hierarchical and complex designs, identification of the appropriate level for tests and full reporting of outcomes
☐	☒	Estimates of effect sizes (e.g. Cohen's *d*, Pearson's *r*), indicating how they were calculated

Our web collection on statistics for biologists contains articles on many of the points above.

Software and code

Policy information about availability of computer code

Data collection	No software was used for data collection.

Data analysis	RNA-SEQ: cudadapt (1.18) STAR (v. 2.7.3a) deepTools (v3.1.0). subread (1.6.5) DESeq2 (1.26.0)<br>CUT&Tag: cutadapt (4.0) bowtie2 (2.4.5) macs2 (2.1.2) GreyListChIP (1.22.0) DiffBind (3.0) deepTools (3.5.1) MEME-ChIP (MEME v. 5.4.1) FIMO (Version 5.4.1)<br>ATAC-seq: cutadapt (4.0) bowtie2 (2.4.5) macs2 (2.1.2) DiffBind (3.6.1) deepTools (v3.5.1) WiggleTools (1.2.8) multiBigwigSummary (Galaxy Version 3.5.1.0.0.)<br>Gviz 1.34.1<br>IGV 2.8.9<br>Mass Spectrometry: Andromeda search engine of the MaxQuant software suite v1.6.5.0<br>Microscopy: VisiView 5.0 software, Imaris (v. 9.9.1)<br>Western Blot & Gels: Image Lab Software Version 6.1<br>SEC-MALS: ASTRA 8 software (Wyatt Technology)<br>Fluorescence Polarization: GraphPad Prism 8<br>Sequence analyses, alignments and evolution: Jalview, Version: 2.11.2.3, MEGA software (version 7.0), Clustal Omega on https://www.ebi.ac.uk/Tools/msa/clustalo/ (no version stated) , Prediction of Intrinsically Unstructured Proteins on https://iupred2a.elte.hu/ (IUPred2A version), DNA binding site predictor on http://zf.princeton.edu/ (no version stated), Nuclear Localization Signal prediction on https://nls-mapper.iab.keio.ac.jp/cgi-bin/NLS_Mapper_form.cgi (no version stated, Last Update: 2012/11/7); Alignment visualization with ESPript available online on https://espript.ibcp.fr/ESPript/ESPript/<br>Statistics & Plotting: R StudioVersion 1.4.1717 with R version 4.1.1<br>Figures: Adobe Illustrator & Photoshop 2021

For manuscripts utilizing custom algorithms or software that are central to the research but not yet described in published literature, software must be made available to editors and reviewers. We strongly encourage code deposition in a community repository (e.g. GitHub). See the Nature Portfolio guidelines for submitting code & software for further information.

Data

Policy information about availability of data

All manuscripts must include a data availability statement. This statement should provide the following information, where applicable:

- Accession codes, unique identifiers, or web links for publicly available datasets
- A description of any restrictions on data availability
- For clinical datasets or third party data, please ensure that the statement adheres to our policy

No restrictions apply and all data is available in the manuscript or the supplementary materials. RNA-seq, CUT&Tag and ATAC-seq data have been deposited to GEO (GSE210624, GSE210630). Mass Spectrometry has been deposited to ProteomeXchange via the PRIDE database (project ID PXD042353). DNA and protein sequences are publicly available and were retrieved from VectorBase (www.vectorbase.org). The ensembl AgamP4 genome using the Ensembl AgamP4 annotation (release 48) was also retrieved via vectorbase.org. Metazoan Upstream Sequences for Anopheles gambiae (AgamP4.34_2019-03-11) or Aedes aegypti (AaegL3.34_2019-03-11) databases used in FIMO are publicly available / selectable under https://meme-suite.org/meme/tools/fimo. RNA-seq data from Papa et al. is publicly available in the Sequence Read Archive (SRA; http://www.ncbi.nlm.nih.gov/sra) under accession number SRP083856.

Human research participants

Policy information about studies involving human research participants and Sex and Gender in Research.

Reporting on sex and gender	This study does not involve Human Research Participants.
Population characteristics	This study does not involve Human Research Participants.
Recruitment	This study does not involve Human Research Participants.
Ethics oversight	This study does not involve Human Research Participants.

Note that full information on the approval of the study protocol must also be provided in the manuscript.

Field-specific reporting

Please select the one below that is the best fit for your research. If you are not sure, read the appropriate sections before making your selection.

☒ Life sciences ☐ Behavioural & social sciences ☐ Ecological, evolutionary & environmental sciences

For a reference copy of the document with all sections, see nature.com/documents/nr-reporting-summary-flat.pdf

Life sciences study design

All studies must disclose on these points even when the disclosure is negative.

Sample size	Sample size, number of replicates, error bars and statistical tests were chosen based on accepted practices in the field, which are stated in Figure legends and methods. Generally experiments were performed independently and reproduced with at least 3 biological replicates. For

	genome-wide datasets we followed the recommendations and practices of the ENCODE and modENCODE consortia (PMID: 22955991). Examples of field specific studies: PMID 32510132, 28457869, 29562179
Data exclusions	No data was excluded.
Replication	Each experiment was repeated at least three times with similar results, unless otherwise noted. All attempts of replication were successful. Details are provided in Figure legends.
Randomization	Samples were allocated to study groups by genotype. No randomization was applied.
Blinding	Investigators were not blinded. Blinding was not relevent as of objective experimental readouts (molecular analysis, visible phenotype) allowing sample allocation.

Reporting for specific materials, systems and methods

We require information from authors about some types of materials, experimental systems and methods used in many studies. Here, indicate whether each material, system or method listed is relevant to your study. If you are not sure if a list item applies to your research, read the appropriate section before selecting a response.

Materials & experimental systems

n/a	Involved in the study	
☐	☒	Antibodies
☐	☒	Eukaryotic cell lines
☒	☐	Palaeontology and archaeology
☐	☒	Animals and other organisms
☒	☐	Clinical data
☒	☐	Dual use research of concern

Methods

n/a	Involved in the study	
☒	☐	ChIP-seq
☒	☐	Flow cytometry
☒	☐	MRI-based neuroimaging

Antibodies

Antibodies used

Anti-SOA Antibody (Custom (Eurogentec), epitope-purified by the IMB PPCF,N.A. (Rabbit 87), #540887-22062021)
Anti-HA.11 Antibody (Biolegend, BLD-901502), clone 16B12
Anti-Histone H3 Antibody (Cell Signalling, 9715S)
Anti-Histone H3 (mAb) Active Motif 39763, clone MABI 0301
RNA pol II antibody (mAb) Active Motif 39097, clone 4H8
RNA pol II CTD phospho Ser2 antibody (mAb) Active Motif 61984, clone 3E10
phospho H3 (S10)Mouse IgG2b, κ Biolegend 650801, clone 11D8
IgG control Antibody (Abcam, ab37415)
Anti-αMs IgG Antibody (Abcam, ab6709)
Anti-Rb IgG Antibody (Sigma-Aldrich, SAB3700377)
Anti-Rb IgG coupled to AF555 Antibody (ThermoFisher, A21430)
Anti-Mouse IgG (H+L) Antibody (Jackson ImmunoResearch, JIM-715-035-150)
Anti-Rabbit IgG (H+L) Antibody (Jackson ImmunoResearch, JIM-711-035-152)

Validation

PRIMARY ANTIBODIES:

SOA antibody was generated in this study. The validations are presented in the paper:
In CUT&Tag (Fig. 2 and 4), SOA mutants (SOA-KI) and non-specific isotype antibody (IgG) controls validate the specific enrichments.
In Immunofluorescence (Fig. 1 and 4), lack of signal in mutants (SOA-KI) and females validate the specificity.
In Western blot (Extended Data Fig. 4e,), the specific immunoprecipitation of SOA, but not control - expressing cells, validate the specificity, which is also confirmed by mass spectrometry (Fig. 1). Detected bands run at the expected molecular weights.

Anti-HA.11 Antibody (Biolegend, BLD-901502) - this study: validated in the western blot and CUT&Tag by using a negative control condition (empty baculovirus). No western blot signal in the empty control; no CUT&Tag peaks in the empty control. Band with expected molecular weight observed in the HA-SOA1-229 expressing sample.
Cited in 424 peer-reviewed article as of 09.12.2022.
Monoclonal antibody against the YPYDVPDYA peptide. Search for this peptide in the A.gambiae proteome yielded no identical sequences.
Validated by the manufacturer for use in western blot.
https://www.biolegend.com/en-gb/products/purified-anti-ha-11-epitope-tag-antibody-11374
“Additional tested and reported applications of the 16B12 clone for the relevant formats include: western blot (WB), immunocytochemistry (ICC), immunoprecipitation (IP), and flow cytometry (FC).”

Anti-Histone H3 Antibody (Cell Signalling, 9715S) - in this study: detected at correct molecular weight (~17 kDa) in the nuclear fraction.
https://www.cellsignal.com/products/primary-antibodies/histone-h3-antibody/9715
Cited in 957 peer-reviewed article as of 12.09.2022 “For western blots, incubate membrane with diluted primary antibody in 5% w/v nonfat dry milk, 1X TBS, 0.1% Tween® 20 at 4°C with gentle shaking, overnight.”

It has been successfully used in in Drosophila (https://www.ncbi.nlm.nih.gov/pmc/articles/PMC4008575/) and H3 is highly conserved.

Anti-Histone H3 (mAb) Active Motif 39763 - in this study: detected in correct nuclear compartment in IF
RRID:AB_2650522. Clone:MABI 0301
Applications Validated by Active Motif:
ChIP-Seq: 4 µg per ChIP
ChIP: 5 - 10 µg per ChIP
ICC/IF: 1 µg/ml dilution
WB: 0.5 - 2 µg/ml dilution
25 publications using antibody on Active Motif Website since 2001

RNA pol II antibody (mAb) Active Motif 39097 - in this study: detected in correct localization (nucleus/chromatin) in IF and Western blot, correct size in Western blot.
RRID:AB_2732926. 12 citations
Applications Validated by Active Motif:
ChIP: 10 µl per ChIP
ChIP-Seq: 20 µl each
WB: 1:2,000 - 1:5,000 dilution
The following applications have been published using this antibody. Unless noted above, Active Motif may not have validated the antibody for use in these applications:CUT&TagC hIP-Seq, ChIP-qPCR ICC/IF WB
24 publications using antibody on Active Motif Website since 2001

RNA pol II CTD phospho Ser2 antibody (mAb) Active Motif 61984 - in this study: correct pattern in IF
RRID:AB_2687450. 12 citations
RRID:AB_2687450
Clone:3E10
Applications Validated by Active Motif:
WB*: 0.5 - 2 µg/ml
ChIP: 20 µg per ChIP
ChIP-Seq: 20 µg each
IF: 1:500 dilution

phospho H3 (S10)Mouse IgG2b, κ Biolegend 650801 - in this study: correct pattern in IF - specific presence in mitotic cells
RRIDAB_10896911 (BioLegend Cat. No. 650801)
AB_10900065 (BioLegend Cat. No. 650802)
Antigen References
1. Choi HS, et al. 2005. J. Biol. Chem. 280:13545.
2. Goto H, et al. 2002. Genes Cells 7:11.
3. Garcia BA, et al. 2005. Biochemistry 44:13202.
4. Hans F, et al. 2001. Oncogene 20:3021.
Product Citations
Friedman J, et al. 2018. J Immunother Cancer. 6:59. PubMed
Han G, et al. 2018. Nat Protoc. 2.014583333. PubMed
IgG control Antibody (Abcam, ab37415)
https://www.abcam.com/rabbit-igg-monoclonal-epr25a-isotype-control-ab172730.html#lb
Shows only background signal in Cut&Tag (this study)
Validated for a similar application (CUT&RUN): "ChIC/CUT&RUN-seq Use 0.5-2µg for 105 cells."
Cited in 302 peer-reviewed article as of 12.09.2022

SECONDARY ANTIBODIES:

Anti-αMs IgG Antibody (Abcam, ab6709)
https://www.abcam.com/rabbit-mouse-igg-hl-ab6709.html
Cited in 33 peer-reviewed article as of 12.09.2022
Affinity purified: "this product was prepared from monospecific antiserum by immunoaffinity chromatography using Mouse IgG coupled to agarose beads"
Successfully used in peer-reviewed studies for CUT&Tag:
"CUT&Tag was performed with CUT&Tag-IT Assay Kit (53160, ACTIVE MOTIF) in 1.5×106 FaDu cells using anti-SF3B2 (sc-514976, Santa Cruz, 5 µL, 1:20 dilution) and anti-H3 (ab1791, Abcam, 1 µL, 1:100 dilution) antibodies. Rabbit anti-mouse IgG (ab6709, Abcam, 1 µL) was used to enhance the signal. The cells were collected using a cell scraper." (https://cellandbioscience.biomedcentral.com/articles/10.1186/s13578-022-00812-8)
"Secondary antibody (Rabbit Anti-Mouse IgG H&L: abcam, ab6709) was diluted 1:100 in dig wash buffer and cells were incubated at RT for 60 min." (https://www.sciencedirect.com/science/article/pii/S0304383521006170)

Anti-Rb IgG Antibody (Sigma-Aldrich, SAB3700890)
The specificity for rabbit immunoglobulins was validated by the manufacturer
https://www.sigmaaldrich.com/DE/en/product/sigma/sab3700890
"This product was prepared from monospecific antiserum by immunoaffinity chromatography using Rabbit IgG coupled to agarose beads followed by solid phase adsorption(s) to remove any unwanted reactivities. Assay by immunoelectrophoresis resulted in a single precipitin arc against Anti-Guinea Pig Serum, Rabbit IgG and Rabbit Serum. No reaction was observed against Goat, Human and Mouse Serum Proteins."

Anti-Rb IgG coupled to AF555 Antibody (ThermoFisher, A21430)
Cited in 125 peer-reviewed article as of 12.09.2022
The specificity for rabbit immunoglobulins in IF was validated by the manufacturer.

https://www.thermofisher.com/antibody/product/Goat-anti-Rabbit-IgG-H-L-Cross-Adsorbed-Secondary-Antibody-Polyclonal/A-21430
"F(ab')2-Goat anti-Rabbit IgG (H+L) Secondary Antibody, Alexa Fluor 555 was used at concentration of 4µg/mL in phosphate buffered saline containing 0.2 % BSA for 45 minutes at room temperature. [...] No nonspecific staining was observed with the secondary antibody alone (panel f), or with an isotype control (panel e)."

Anti-Mouse IgG (H+L) Antibody (Jackson ImmunoResearch, JIM-715-035-150)
Cited in 534 peer-reviewed article as of 12.09.2022
The specificity for mouse immunoglobulins was validated by the manufacturer
https://www.jacksonimmuno.com/catalog/products/715-035-150
"Based on immunoelectrophoresis and/or ELISA, the antibody reacts with whole molecule mouse IgG. It also reacts with the light chains of other mouse immunoglobulins. No antibody was detected against non-immunoglobulin serum proteins."
"Suggested Working Concentration or Dilution Range: 1:10,000 - 1:200,000 for Western blotting with ECL substrates"

Anti-Rabbit IgG (H+L) Antibody (Jackson ImmunoResearch, JIM-711-035-152)
Cited in 845 peer-reviewed article as of 12.09.2022
The specificity for rabbit immunoglobulins was validated by the manufacturer
https://www.jacksonimmuno.com/catalog/products/711-035-152
"Based on immunoelectrophoresis and/or ELISA, the antibody reacts with whole molecule rabbit IgG. It also reacts with the light chains of other rabbit immunoglobulins. No antibody was detected against non-immunoglobulin serum proteins."
"Suggested Working Concentration or Dilution Range: 1:10,000 - 1:200,000 for Western blotting with ECL substrates"
Successfully used in a closely related dipteran species, Drosophila melanogaster in western blot applications (3 peer-reviewed citations)

Eukaryotic cell lines

Policy information about cell lines and Sex and Gender in Research

Cell line source(s)	AG55 cells were kindly provided by Prof. Mike Adang.
Authentication	Cell lines were authenticated by RNA-seq.
Mycoplasma contamination	Cells were regularly tested for mycoplasma (MycoAlert PLUS Mycoplasma Detection Kit, Lonza LT07-701). All test were negative.
Commonly misidentified lines (See ICLAC register)	No commonly misidentified cell lines were used in this study

Animals and other research organisms

Policy information about studies involving animals; ARRIVE guidelines recommended for reporting animal research, and Sex and Gender in Research

Laboratory animals	Mosquitos were blood-fed with CD-1 mice of both sexes aged 8-56 weeks. Mice were maintained in social groups of 4-5 individuals with 12h/12h dark/light cycle, 22°C temperature and 50 +/- 10% humidity.<br><br>Anopheles: Pupae used for RNAseq were collected 12h after pupariation. Adult mosquitoes used for immunostainings were 2 - 5 days old. For embryos, different stages (hours after oviposition) were studied, which is specified precisely in the respective Figures and legends.
Wild animals	The study did not involve wild animals.
Reporting on sex	Not relevant, since laboratory animals were only used for blood-feeding of mosquitos, which is required for female egg laying / husbandry.
Field-collected samples	The study did not involve field-collected samples
Ethics oversight	We have complied with all relevant ethical regulations regarding the use of animals for this project authorized by the French ministry of higher education, research and innovation under the number APAFIS#20562- 2019050313288887 v3.

Note that full information on the approval of the study protocol must also be provided in the manuscript.
